# PhyCysID: Plant Cystatin Protein Prediction by an Artificial Intelligence Approach

**DOI:** 10.1021/acs.jcim.5c00916

**Published:** 2025-09-04

**Authors:** Sadaf Aqil, Isabel C. Cadavid, Nureyev F. Rodrigues, Natalia Balbinott, Geancarlo Zanatta, Rogerio Margis

**Affiliations:** † Programa de Pós-Graduação em Genética e Biologia Molecular, Departamento de Genética, Instituto de Biociências, 541470 Universidade Federal do Rio Grande do Sul, Porto Alegre CEP 91501-970, Brazil; ‡ Departamento de Biofísica, Centro de Biotecnologia, Universidade Federal do Rio Grande do Sul, prédio 43422, sala 206, Porto Alegre CEP 91501-970, Brazil

## Abstract

Phytocystatins are proteinaceous inhibitors found in plants that competitively target various classes of cysteine proteinases, including papain-like enzymes, cathepsins, and legumains. Based on structural characteristics and gene organization, phytocystatins can be classified into four subtypes: intronless (I1 and I2), intron-containing (IwI), and multidomain cystatins containing more than one inhibitory region (II). This work presents PhyCysID, a dedicated web server designed for the rapid classification of phytocystatin subtypes. PhyCysID uses a set of 21 features derived from amino acid composition, in combination with 15 distinct machine learning algorithms, to classify phytocystatin sequences into one of the four subtypes. Initially, the input sequence is analyzed to verify if it comprises a true phytocystatin sequence. If so, the input sequence is further analyzed using a specialized classification pipeline called PhyCysID 12M, which integrates 12 machine learning models to assign it to one of the four defined phytocystatin classes. As a case study, a curated dataset of phytocystatin sequences from the UniProt database was used to evaluate the algorithm’s performance. The PhyCysID web server enables rapid classification of both individual and batch-submitted sequences in less than 15 s, providing high-throughput analysis for an accurate identification of phytocystatin class and function. PhyCysID is freely available at https://www.ufrgs.br/labec/phycysid.

## Graphical abstract

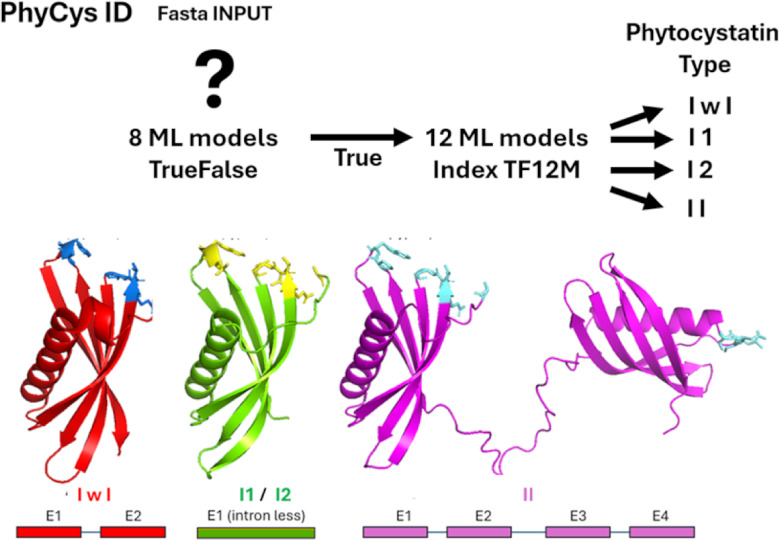

## 1 Introduction

Phytocystatins (PhyCys) or plant cystatins, as their animal counterparts, are strong inhibitors of cysteine proteinases. They control a number of physiological responses, such as senescence,^1^ seed germination^2^ and have a part in plant growth and development and are important for responding to biotic and abiotic stresses.^3^ PhyCys regulate both endogenous and exogenous proteases in a functional capacity. Besides acting on the regulation of proteolysis to maintain protein homeostasis, phytocystatins also inhibit exogenous cysteine proteases from pathogens and herbivorous^4,5^ and hinder the growth of bacteria, protozoa, and fungal pathogens.^6^ Moreover, phytophagous insects are adversely affected when their digestive cysteine proteases are blocked, which lowers protein digestion and impairs their ability to develop and survive.^7^ In addition to their role in biotic stress responses, phytocystatins also have a role in abiotic stress reactions. For instance, the overexpression of MpCYS4 in apples postponed stress-induced and natural leaf senescence and alleviated the associated oxidative damage.^1^ Similarly, overexpressing AtCYS5 in *Arabidopsis* improved heat stress tolerance.^8^

Evolutionarily, phytocystatins are well conserved among plants and animals. The cystatin domain is characterized by three motifs essential to the inhibitory function: a glycine residue in the protein flexible amino-terminal region, a QxVxG motif in the central region and a conserved [A,P]-W residue near the carboxy-terminal region.^3^ Cystatins share a conserved protein folding of five antiparallel beta sheets surrounding a central α-helix. The inhibitory domains are located in the loops resulting from this structure. The primary targets of phytocystatins are cysteine proteinases similar to papain-like proteinases (family C1, MEROPS). However, a smaller group of phytocystatins is also capable of inhibiting legumain-like proteinases (family C13, MEROPS) and have a second conserved motif characterized by the sequence S–N–S-[L,I].^9^ Comparative studies of plant cystatin structures revealed a unique motif in the amino-terminal α-helix, specifically [LVI]-[AGT]-[RKE]-[FY]-[AS]-[VI]-x-[EDQV]-[HYFQ]-N, which is found exclusively in plant cystatins. To date, this structural feature has not been associated with any specific function.^10^

Typically, phytocystatins are distinguished by their molecular weight, structural arrangement (Figure 1) and phylogenetic relationships. Type-I phytocystatins (PhyCys-I) with a molecular weight that ranges from about 10 to 16 kDa, have a single inhibitory domain for papain-like proteases and display the traditional cystatin fold. Type-II phytocystatins or PhyCys-II, have an extra carboxy-terminal extension that takes on a fold that resembles the standard single-domain cystatin structure. These proteins have inhibitory capacities toward both papain-like and legumain-like proteases through the activity of motifs present in their amino- and carboxy-terminal regions, respectively (Figure 1C). Their molecular weight rises to roughly 23–26 kDa as a result of this duplication. Since PhyCys-II structural organization has not been found in any other organism, it seems to be exclusive to plants. Another group of phytocystatins is classified as Type III or multicystatins, characterized by 2 to 8 duplications of the papain-type proteinase inhibitory region. This subtype is less frequent and primarily occurs in members of the Solanaceae family, with the duplications being phylogenetically related to Type-I intron-containing phytocystatins (IwI).

**Figure 1:**
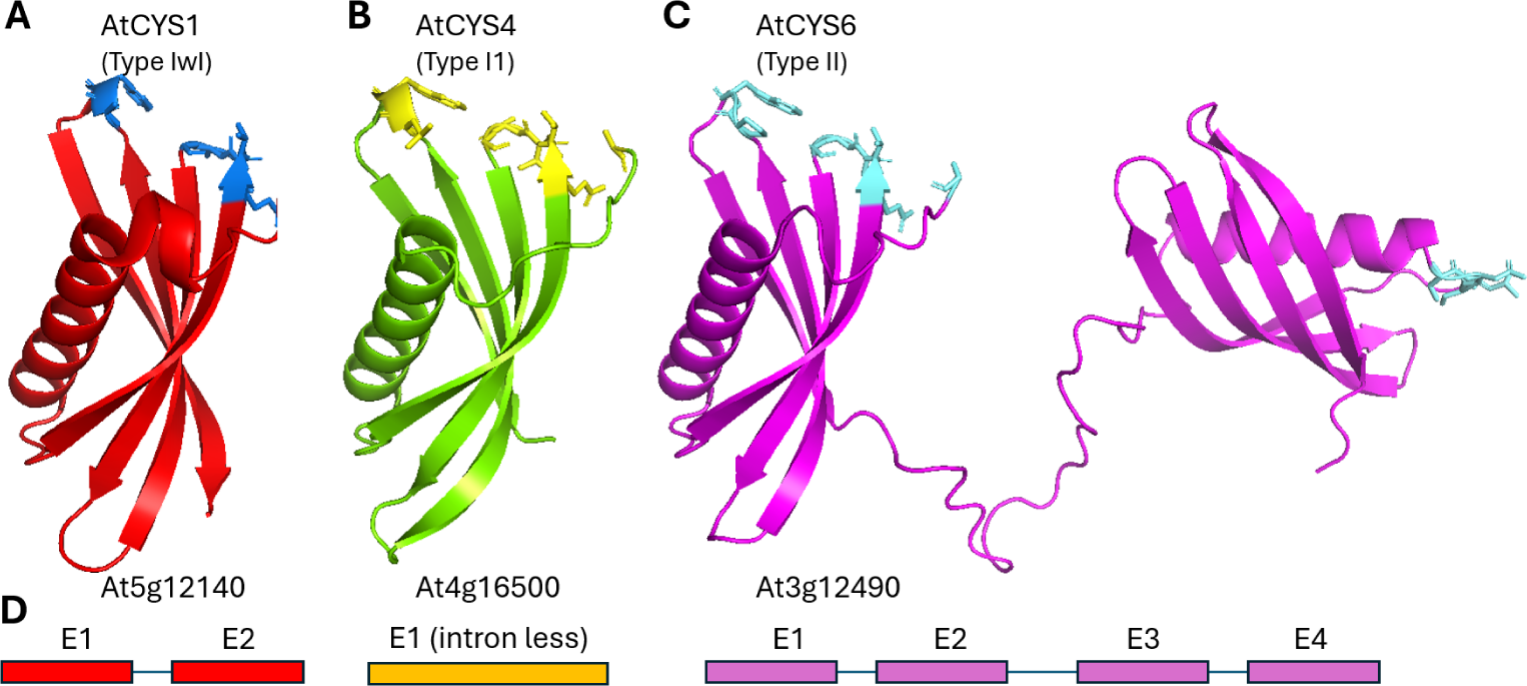
3D structure of each of the three genomic paralog forms of *Arabidopsis thaliana* phytocystatins. A) Type-I phytocystatin with intron, B) type-I intron-less phytocystatin, and C) type-II phytocystatin with intron and carboxy-terminal legumain inhibitory domain. Residues corresponding to the QxVxG and xW papain inhibitory motifs and SNSL legumain inhibitory motifs are represented as sticks and with different colors. D) Genomic organization of phytocystatin paralogs.

Another possible classification of phytocystatins is according to their gene structure, more specifically by the presence or absence of introns. PhyCys-II genes generally have a consistent structure featuring four exons and three introns (Figure 1D). The first two exons encode the papain-like inhibitory domain, while the last two exons encode the legumain-like inhibitory domain. In contrast, PhyCys-I genes may be intronless, which is more common in flowering plants, or they may have two or more exons, with the two-exon configuration being the most prevalent.^11^

Using phylogenetic analysis, the evolutionary processes of phytocystatins were better understood by identifying unique clusters.^12^ Three primary clusters were revealed: one that contains PhyCys-I and PhyCys-II from species spanning Viridiplantae and two of which are mainly made of PhyCys-I from Angiosperms. Interestingly, phytocystatins with two or more exons are represented by the majority of sequences in the first cluster (Figure 2). On the other hand, the last two clusters, I1 and I2, are primarily represented by Angiosperm genes that lack introns, while a minor fraction of sequences have one intron.

**Figure 2:**
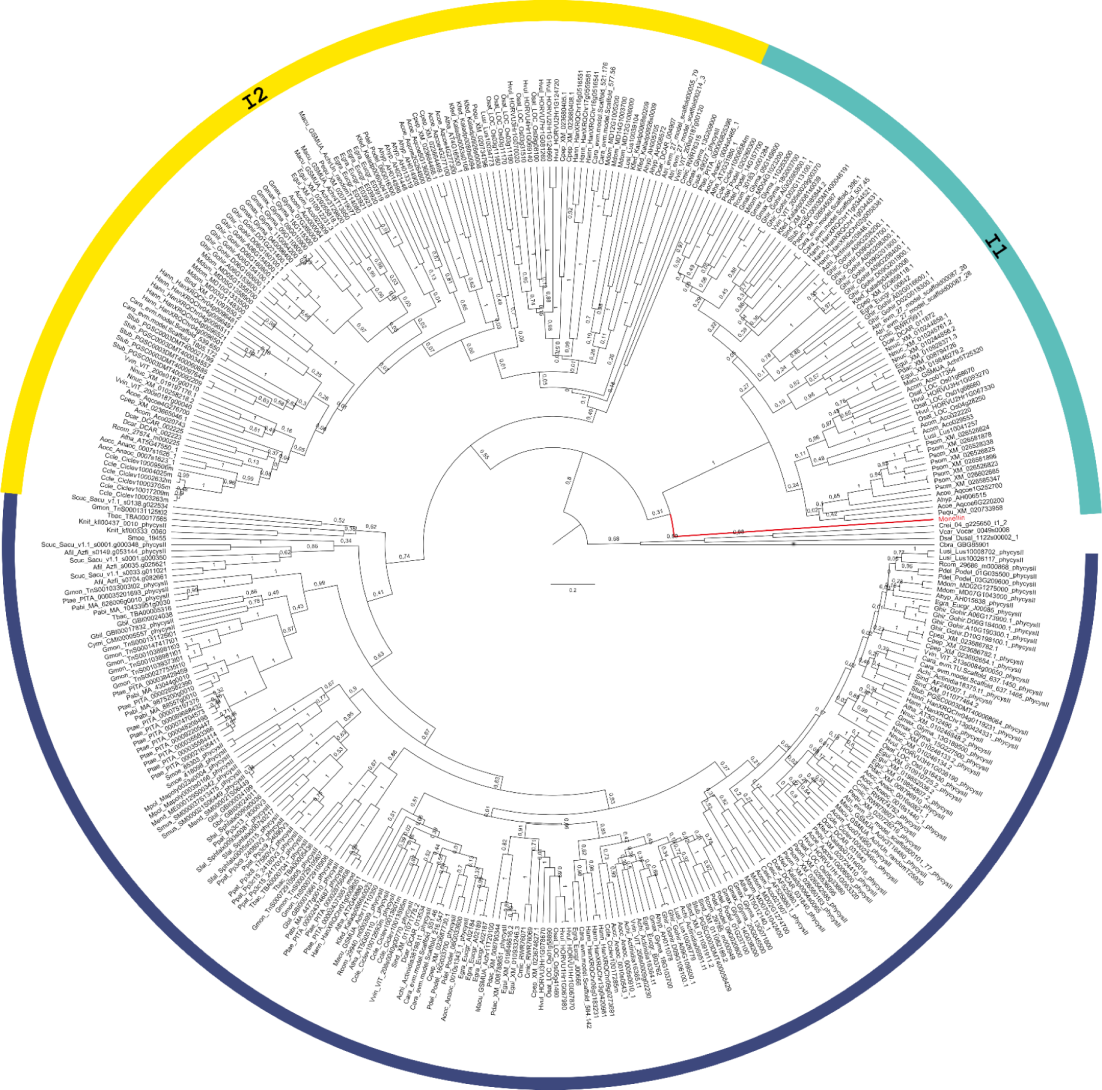
Phylogenetic tree depicting the evolutionary relationships among plant cystatins and monellin, based on protein sequences. Intronless 1 (I1) and 2 (I2) branches are colored in turquoise and yellow, respectively, while type I with intron (IwI) and type-II branches are in the region corresponding to dark-blue circle, and monellin branch is colored in red. Tree was constructed using Bayesian Inference with BEAST2. Posterior probabilities are indicated at the nodes.

The amino-terminal regions of PhyCys-I and PhyCys-II exhibit significant sequence conservation, especially in areas corresponding to the inhibitory domain and the interaction sites between α-helices and β-sheets, which are crucial for maintaining protein structure. Amino acid residues within the α-helix and β-sheets are either highly conserved or show conservative substitutions that do not substantially change the charge or size of the amino acids. Regarding the LARFAVDEHN motif, which is a hallmark of plant cystatins, there is greater conservation in phytocystatins with introns, whereas intronless phytocystatins tend to have more sequence variability.^12^

Interestingly, some proteins outside the phytocystatin subfamily share structural similarities with its members, despite lacking any protease inhibitory capacity. Monellin, often misclassified as a phytocystatin, is a small sweet tasting protein whose structural features resemble those of phytocystatins.^13^ It was discovered in 1969 in the fruit of the West African shrub known as the serendipity berry and no gene model is predicted for it to this date. Research confirmed that monellin is indeed a protein with a molecular weight of around 10–10.5 kDa and is composed solely of amino acids, without any detectable carbohydrate or fatty acid residues. A remarkable feature of monellin is that, in certain analyses such as molecular weight determination and impurity checks, it has initially appeared to be a single polypeptide chain.^14^ However, sequencing analysis revealed that two amino acids were released at each degradation step, suggesting the presence of two distinct polypeptide chains that degrade simultaneously.^15^ Further by separating the chains using an organomercurial agarose column, monellin gives a unique structure made of the two chains.^16^ Although the structure of monellin has been well characterized and shows notable similarity to phytocystatins, no corresponding gene model has been annotated and no homologues have been identified in other plant species, indicating that monellin may be unique to serendipity berry.

The evolutionary history of phytocystatins highlights both structural conservation and sequence diversification across plant lineages.^12^ The most recent common ancestor (MRCA) of the Viridiplantae family suffered tandem duplication of an ancestral cystatin-like gene, resulting in a carboxy-extended cystatin variant that is specific to plants. The current form of PhyCys-II evolved from this carboxy extension by neofunctionalization and the acquisition of an inhibitory capacity against legumain-like proteases. As the carboxy-extended version of phytocystatin developed, the original single-domain phytocystatin also changed, giving rise to a portion of the present PhyCys-I containing introns. A single-domain phytocystatin underwent retroduplication in the MRCA of flowering plants, which led to intron loss and the formation of intronless PhyCys-I. Then, the I1 and I2 clusters were formed by the duplication and diversification of this original form. Multiple rounds of gene duplication in both PhyCys-I and PhyCys-II have generated the current diversity and species-specific expansion, including the appearance of PhyCys-III, throughout the evolutionary history of the family.

The structural and functional diversity among phytocystatin gene models provides a foundation for developing computational approaches capable of classifying these proteins based on their sequence analysis. Gene prediction has advanced significantly from basic rule-based methods to sophisticated computational models that integrate machine learning and artificial intelligence. Early techniques relied on sequence homology and heuristic rules,^17^ while contemporary approaches utilize advanced statistical models, RNA-Seq data, and deep learning algorithms.^18,19^ By integrating various methodologies and diverse data sources, researchers can achieve precise predictions of gene locations, structures, and functions, thereby deepening our understanding of genomics and biology.

In the context of plant cystatin genes, accurate gene prediction is crucial for applications in biotechnology and agriculture. Reliable identification and classification of phytocystatins enable targeted functional studies, facilitate and support crop improvement strategies. To address this need, we developed an artificial intelligence-based web server, named PhyCysID, specifically designed to predict intronless cystatin genes. Through a user-friendly interface, the PhyCysID web server leverages the power of artificial intelligence algorithms to quickly and accurately classify input sequences into four different gene classes: Type-I with intron (IwI), intronless Type-I I1 and I2, and Type II.

## 2 Materials and Methods

### 2.1 Phylogenetic Analysis

Phytocystatin protein sequences were retrieved from public genome and transcriptome databases using BLASTp searches against *Arabidopsis* reference sequences. In total, 377 phytocystatin sequences from 53 species from Viridiplantae were used, of which 66 and 112 belong to the group I1 and I2 of intronless phytocystatins, respectively. On top of that, the N-terminal of 83 type-II phytocystatins was also included in the analysis, along with the translation of monellin nucleotide sequence (NCBI identifier JQ282905). Redundant and low-quality sequences were removed and multiple sequence alignment was performed using ClustalW^20^ with MEGA7 ^21^ default settings, followed by manual inspection and refinement. The best-fit protein substitution model was determined using ProtTest 3.4 ^22^ and selected based on the Bayesian Information Criterion (BIC). Phylogenetic analyses were performed using BEAST2 v2.6.3 ^23^ using a birth and death process as the tree prior and a strict log-normal molecular clock model. MCMC analysis was run for 180 million generations, sampling every 10,000 generations. Convergence was assessed using Tracer v1.7.2 ^24^ and the first 10% of sampled trees were discarded as burn-in. A maximum clade credibility (MCC) tree was constructed using TreeAnnotator with median node heights. The resulting phylogenetic tree was visualized and annotated in FigTree v1.4.4.^25^

### 2.2 Featurization Process for Each Input Sequence

Biopython package was employed to extract physicochemical features, and data handling was performed using Pandas and NumPy. A total of 21 features, derived from amino acid residues and their physicochemical properties, were calculated. These include aliphatic content (A, V, I, L, M), net charge at pH 7.4, acidic residues (D, E), hydrophobic aromatic group I (F, Y, W), aromatic or resonance-capable residues (F, Y, W, H), helix propensity, hydrophilicity index, hydrophobicity index, instability index, isoelectric point (pI), strongly basic residues (K, R), basic residues (K, R, H), helix breakers (P, N), N-glycosylation motifs (PNGS), RNA-binding potential, redox potential, all polar residues (S, T, Y, D, E, H, Q, K, R), sequence length, β-sheet propensity, residues containing hydroxyl groups (T, S), and turn-forming propensity. To better understand the differences among the various machine learning models used, a subroutine was also added to calculate the importance of each feature or linear coefficient for each model (Supplementary File 1). The use of the scikit-learn package in Python allows the extraction of feature importances_ values from machine learning models such as Decision Trees, Random Forests, Gradient Boosting, and AdaBoost, while the coef_ attribute was used to calculate feature contributions for Logistic Regression, Ridge, Perceptron, Linear Discriminant Analysis, and MLPClassifier. Machine learning models such as Quadratic Discriminant Analysis, Naive Bayes, and K-Nearest Neighbors do not provide direct attributes for feature importance. The models that provide feature_importances_ directly are Random Forest, where the mean decrease in impurity (Gini importance): average total reduction in impurity brought by each feature over all trees; Gradient Boosting is similar to Random Forest and is based on how often and how effectively a feature is used to reduce impurity across boosting rounds; in AdaBoost the importance comes from how frequently a feature is chosen in the weak learners and the weight (alpha) assigned to them. Other machine learning models, typically based on linear approaches, use a coefficient attribute (coef_) to quantify feature contributions. In Logistic Regression, the coefficients are derived from the logistic function; higher absolute values indicate greater influence of a feature on the prediction. The Ridge Classifier follows a similar calculation but includes L2 regularization, a technique used to prevent overfitting by penalizing large coefficients through the addition of a term proportional to the square of their magnitudes. In the case of the Perceptron, the weight vector is obtained through online learning updates. For the SGD Classifier, the coefficients are learned via stochastic gradient descent and can be interpreted similarly to those in Logistic Regression. In scikit-learn, the coef_ attribute represents the linear decision boundaries and can be used to estimate feature importance. To allow comparison with feature_importances_, the coef_ values were normalized between 0 and 1 after initial calculation.

### 2.3 Training Datasets

For the classification task, models were trained using features calculated from 537 phytocystatin processed protein sequences in order to remove the signal peptide and extra residues until the first glycine from the inhibitory domain (Figure 3). The dataset was divided into a training set (70%) for model learning and a test set (30%) to assess the generalization performance of the model.

**Figure 3:**
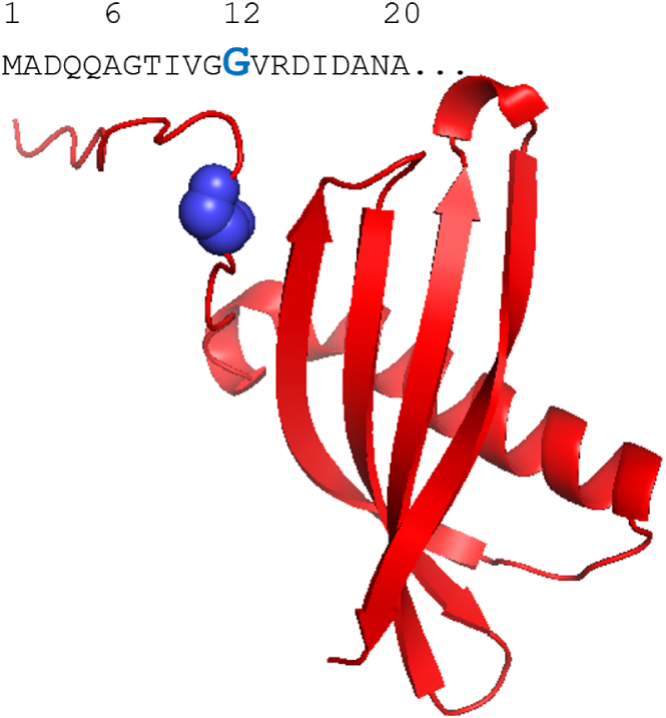
3D structure of AtCYS1 with its amino-terminal extension. The conserved amino-terminal glycine from the inhibitory domain is shown in blue in space-fill format to better illustrate its position relative to the two inhibitory loops. The sequence of the first 20 residues is also displayed to indicate the glycine position along the primary sequence that will be removed by the GG cutter.

A graphical representation of type-I, intron-less type-I and type-II phytocystatins is shown in Figure 1. Figure 3 indicates the regions after the first of the double glycine residues until the end of the testing sequences. A set of proteins not belonging to the phytocystatin subfamily was also used to train the models. This negative set comprises 537 random sequences from the *Arabidopsis thaliana* proteome that do not contain phytocystatin-related domains, each ranging from more than 90 to fewer than 250 amino acids in length. All protein sequences used in the analysis are provided in Supplementary File 2. Sequence identification can be verified via the FASTA header line, which includes an indication of the phytocystatin type (IwI, I1, I2, or II). For nonphytocystatin sequences, the prefix “No_” precedes the gene code position in the *Arabidopsis* genome.

### 2.4 Machine Learning Models

The selected machine learning models encompass diverse classification approaches, ensuring robustness in capturing various data patterns. Linear models assess direct relationships between predictors and target variables, while SVMs handle high-dimensional data with optimized decision margins. Tree-based and ensemble models excel in detecting nonlinear patterns and managing imbalanced datasets, whereas nearest neighbor and probabilistic methods provide intuitive, distribution-based classifications. Neural networks and discriminant analysis enhance flexibility and class separability, while ensemble techniques integrate multiple classifiers to improve the overall performance. In total, 15 machine learning models were tested: Random Forest (RF), Gradient Boosting (GB), AdaBoost, Bagging, Logistic Regression (LR), Gaussian Naive Bayes (GNB), Bernoulli Naive Bayes (BNB), Decision Tree (Decision T), Quadratic Discriminant Analysis (QDA), Linear Discriminant Analysis (LDA), Multilayer Perceptron Classifier (MLPC), K-Nearest Neighbors (K-NN), Ridge Classifier (RC), Perceptron, and Stochastic Gradient Descent Classifier (SDGC). Hyperparameters were set using Grid search technique, and each model underwent cross-validation through 20 iterations. In Supplementary File 3, all possible combinations of hyperparameters for each appropriate ML model were tested and ranked to identify the best performing configuration.

### 2.5 PhyCysID Web Server Interface and Infrastructure

The web server interface was developed using HTML for the front-end, while the code was written in Python and the original code provided in Supplementary File 4. The server machinery employs Flask (https://flask.palletsprojects.com/en/stable/), a micro web framework for Python, to enable communication between the client-side and the underlying Python code. The application is hosted in a 12-core virtual machine in the Data Processing Center (Centro de Processamentos de Dados, CPD) in the Federal University of Rio Grande do Sul.

## 3 Results and Discussion

The PhyCysID web server starts by processing a FASTA file containing protein sequences. Each sequence is computationally scanned for either a diglycine (GG) motif or the first occurrence of a glycine (G) residue within the initial 50 amino acids of the amino-terminal region. Upon detection, the sequence segment downstream to the identified site is extracted for subsequent analyses (Figure 3). This cleavage strategy effectively standardizes the input by focusing on the functional region of each sequence, removing the amino-terminal signal peptide present in some phytocystatins that are targeted to the endoplasmic reticulum, organelles, or secreted via the apoplast. The resulting GG-cleaved FASTA file is then used to generate a feature table encompassing 21 descriptors reflecting the physicochemical and structural properties of the constituent amino acids, which is feeded into the machine learning classification pipeline. Data obtained from each model is then used to calculate the TF8M index and results are made available to the user through the output page. As stated in the PhyCysID pipeline, eight machine learning models are integrated using a majority voting approach. Specifically, each model independently classifies a given input sequence, and the final classification is determined based on the most frequently predicted class across all models. This ensemble method was selected to enhance robustness and minimize model-specific biases. A sequence must be recognized by at least four out of eight machine learning models to be considered a phytocystatin. A detailed visualization of the analysis process is depicted in Figure 4. Due to the complexity of the analysis, the pipeline was divided into two steps: (i) the identification of phytocystatin sequences, and (ii) the classification of each sequence into phytocystatin sub types.

**Figure 4:**
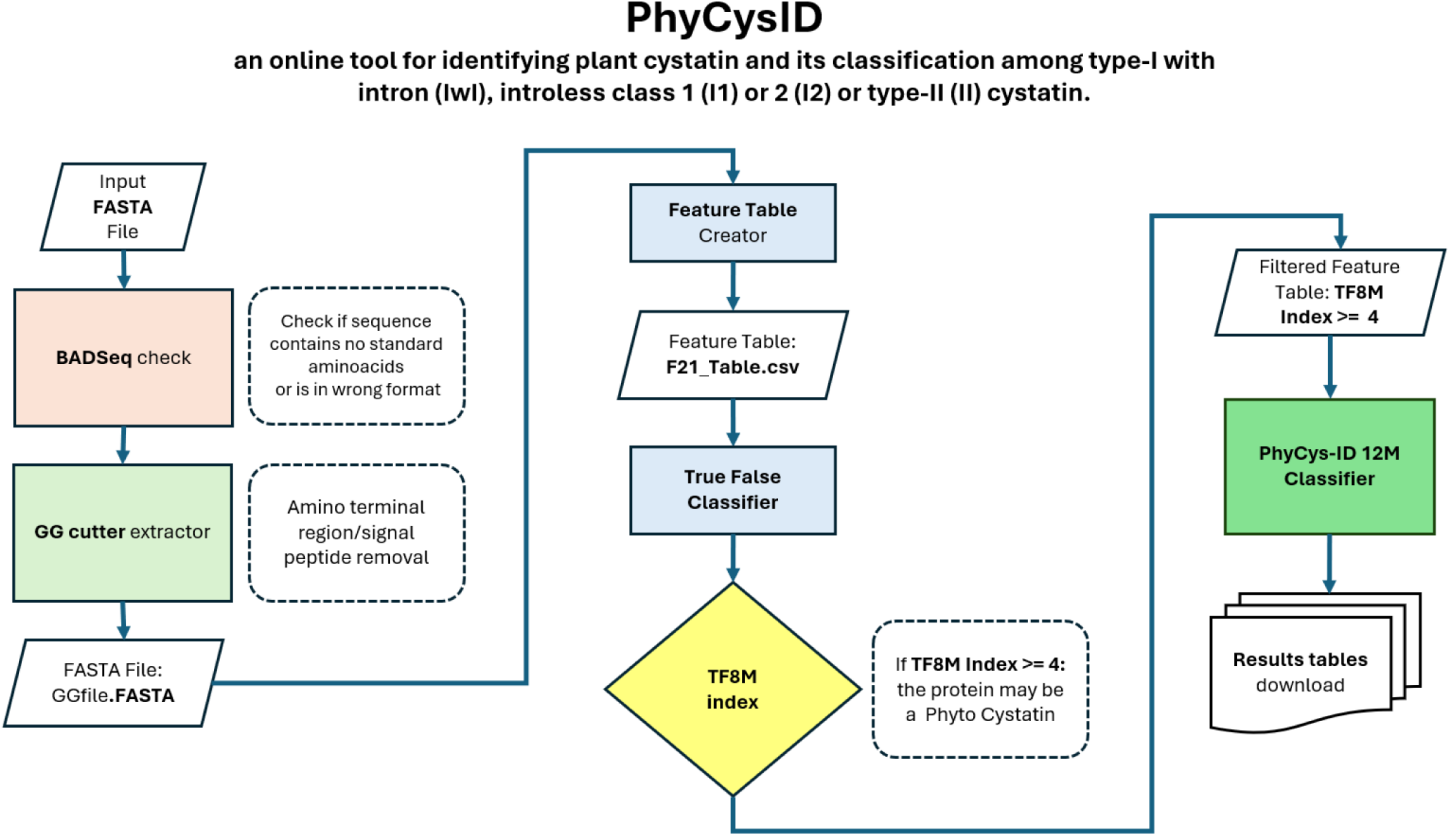
PhyCysID flowchart of data preprocessing, classification and outputs.

In the first step, a dataset consisting of 1,074 sequences, equally divided between TRUE and FALSE phytocystatins, was employed to train each model. During training, multiple machine learning algorithms were tested, including Random Forest, Gradient Boosting, AdaBoost, Bagging, Logistic Regression, K-Nearest Neighbors, MLP Classifier, and Quadratic Discriminant Analysis. For consistency, the training data was normalized using a StandardScaler and the dataset splitted into training and testing sets using a 70/30 ratio with class stratification to ensure balanced learning. Hyperparameter optimization was conducted using GridSearchCV, and robustness was assessed through 20-fold cross-validation.

To gain insight into the analysis, for models that support it, feature importance values (or normalized coefficients) were extracted and compiled in Figure 5. This figure illustrates the relative relevance of each feature within its respective model, emphasizing how each feature contributes to enabling a specific machine learning model to classify and discriminate between TRUE (a cystatin sequence) and FALSE (a noncystatin sequence). A complete list of features, models and values are presented in the Supplementary File 1. It is important to note that the values assigned to each feature by the four models exhibited distinct profiles, suggesting that the different machine learning algorithms possess varying capabilities in classifying true and false phytocystatin sequences and subtypes. Note that while redox potential has a large weight for GB, AB and RF models, it has minor relevance in LR model. The redox potential is reflected by the presence of cysteine residues within the sequences. A detailed analysis of the sequences from the four phytocystatin groups and the outgroup (Supplementary File 2) reveals that the frequency of sequences containing two or more cysteines is only 10.8% among the phytocystatins, whereas it reaches 61.5% in the outgroup proteins. The functional implications of this lower frequency of cysteine residues in phytocystatins remain unknown and are not easily inferred. The only apparent correlation is with the group of animal stefins, which are cystatins that lack disulfide bonds in their structure, in contrast to other animal cystatins that rely on disulfide bridges to maintain their three-dimensional conformation. Sequence size consistently shows high importance across all models, while features like Turn Propensity, TS, and Instability Index exhibit moderate but stable relevance in ensemble methods and LR. Physicochemical properties such as Charge at pH 7.4 and Hydrophobicity Index demonstrate comparable importance in AB and LR, with modest influence in GB. Notably, FYWH, PNGS, and RNA Binding contribute more significantly in LR, which overall presents a more uniform feature importance distribution. Additionally, features like KR, KRH, and STYDEHQKRH maintain moderate importance across models, indicating a supportive role in classification.

**Figure 5:**
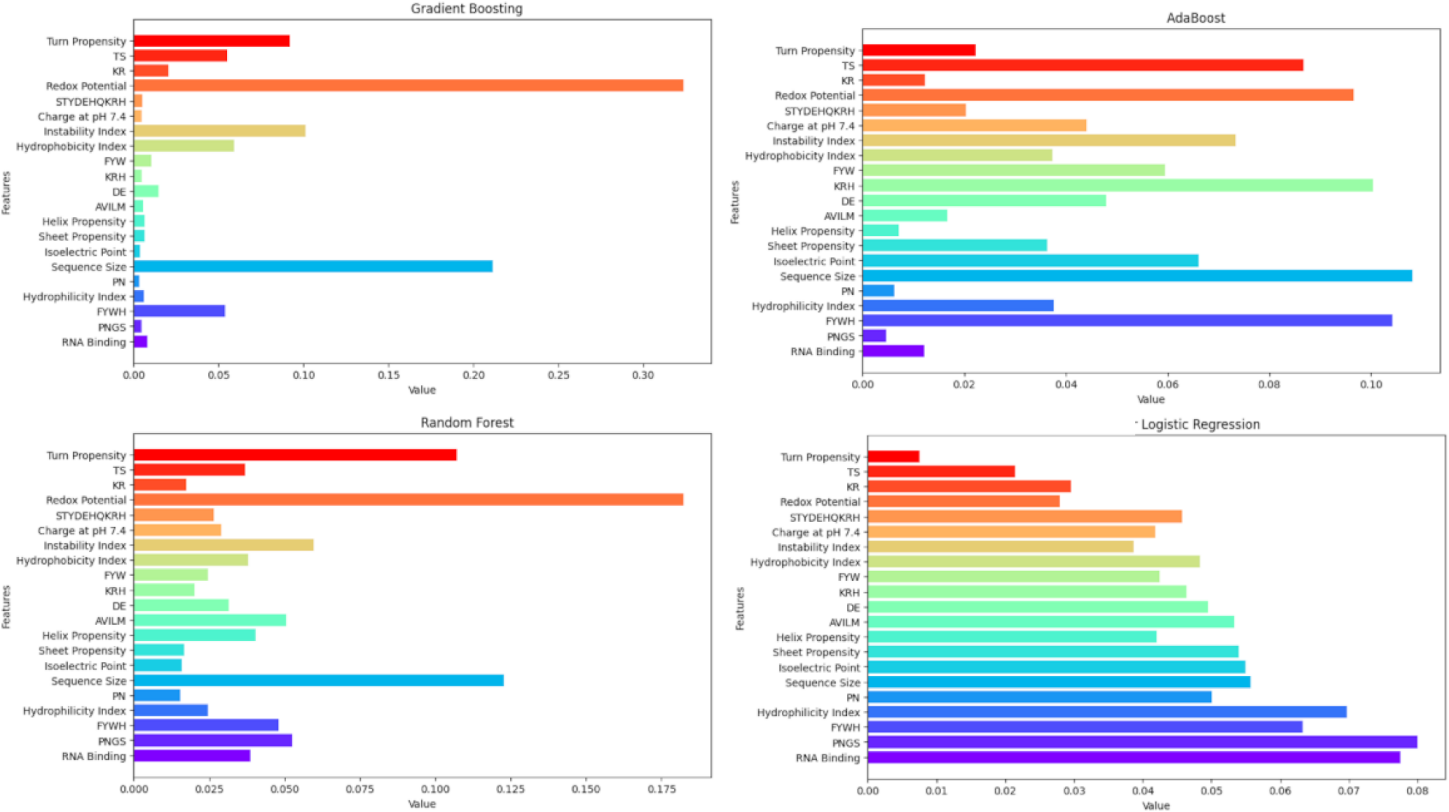
Relative importance of features in four different machine learning models used to calculate the PhyCysID.

For consistency, each model was evaluated through performance metrics such as accuracy, precision, specificity, recall, FPR, F1-score, AUC-ROC, a 20-fold cross-validation and confusion matrix analysis, and only high-performing models showing accuracy above a cutoff value of 0.9 were selected (Figure 6). Surprisingly, different models delivered slightly distinct predictions with overlapping results. To further improve our analysis we investigated the integration of results from each of the eight selected models. Such an approach generated the Index-TF8M, which improved the metrics bringing robustness to the analysis. A general overview of the performance of individual methods in comparison to the Index-TF8M is shown in Table 1.

**Figure 6:**
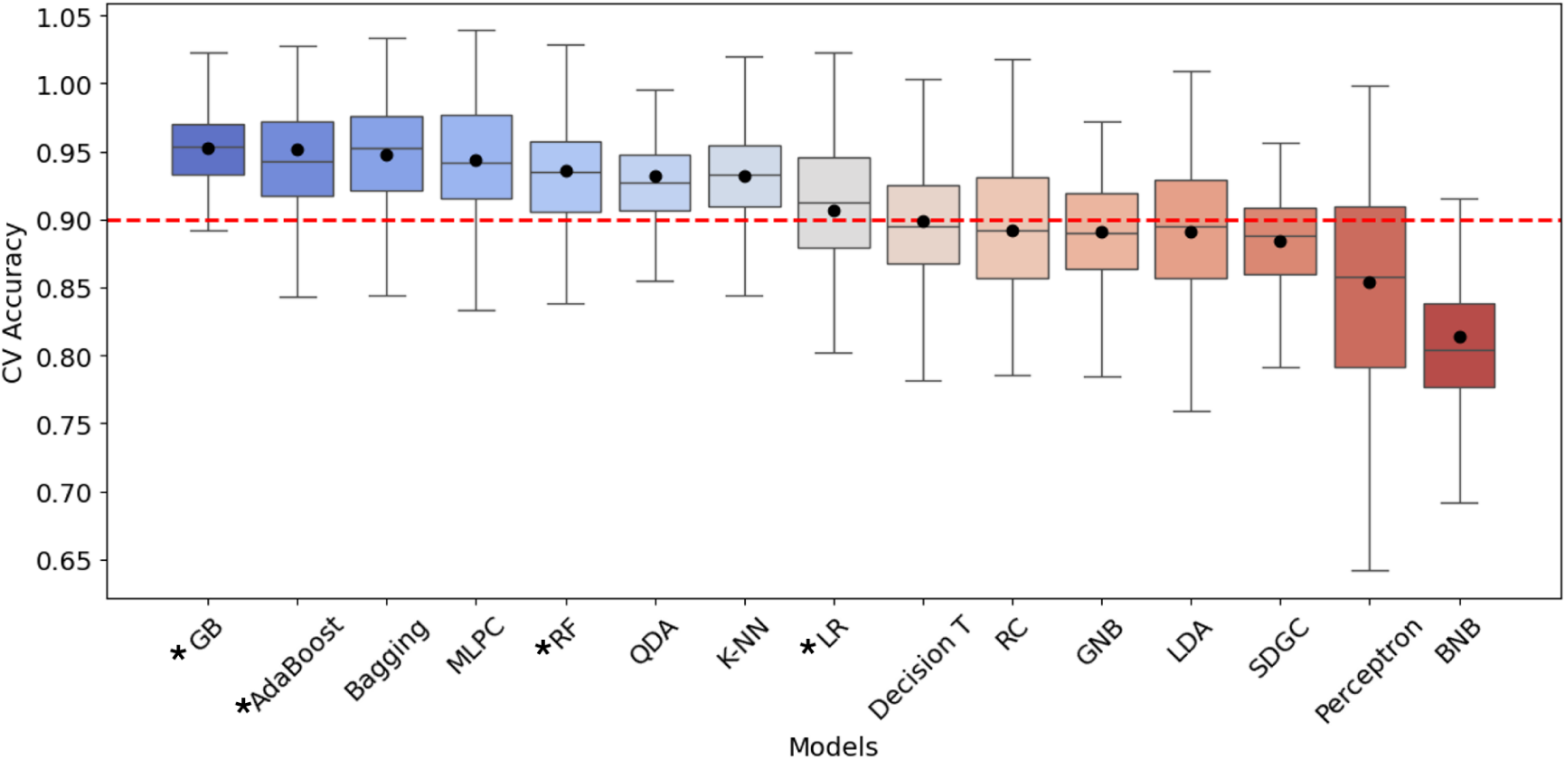
Graphical representation of cross-validation accuracy for the 15 machine learning models. Data are presented as box plots, with black dots indicating mean accuracy. The dotted line marks 0.9 accuracy, used as the cutoff point for calculating the phytocystatin identification index. Asterisks denote models for which the relative importance of each feature was calculated.

**Table 1:** Model Performance Evaluation Matrices for Phytocystatin TRUE or FALSE Prediction

Model	Accuracy	Precision	Specificity	Recall	FPR	F1-Score	AUC—ROC	CV Accuracy	CV Error	Confusion Matrix [TP,FP],[FN,TN]]
**GB**	**0.944**	**0.945**	**0.944**	**0.944**	0.056	**0.944**	**0.984**	**0.952**	0.033	[[151, 11], [7, 154]]
**AdaBoost**	**0.932**	**0.933**	**0.932**	**0.932**	0.068	**0.932**	**0.980**	**0.951**	0.039	[[154, 8], [14, 147]]
**Bagging**	**0.947**	**0.948**	**0.947**	**0.947**	0.053	**0.947**	**0.987**	**0.948**	0.034	[[155, 7], [10, 151]]
**MLPC**	**0.963**	**0.963**	**0.966**	**0.963**	0.034	**0.963**	**0.993**	**0.944**	0.044	([155, 7], [5, 156]]
**RF**	**0.954**	**0.954**	**0.960**	**0.954**	0.040	**0.954**	**0.989**	**0.936**	0.045	[[157, 5], [10, 151]]
**QDA**	**0.929**	**0.930**	**0.929**	**0.929**	0.071	**0.929**	**0.978**	**0.932**	0.032	[[154, 8], [15, 146]]
**K-NN**	**0.901**	**0.907**	**0.901**	**0.901**	0.099	**0.901**	**0.953**	**0.932**	0.039	[[136, 26], [6, 155]]
**LR**	**0.926**	**0.926**	**0.926**	**0.926**	0.074	**0.926**	**0.978**	**0.907**	0.052	[[151, 11], [13, 148]]
Decision T	0.879	0.879	0.876	0.879	0.124	0.879	0.879	0.899	0.053	[[143, 19], [20, 141]]
RC	0.895	0.896	0.895	0.895	0.105	0.895	N/A	0.892	0.053	[[141, 21], [13, 148]]
GNB	0.861	0.861	0.861	0.861	0.139	0.861	**0.953**	0.891	0.050	[[138, 24], [21, 140]]
LDA	0.892	0.892	0.892	0.892	0.108	0.892	**0.963**	0.891	0.054	((141, 21], [14, 147]]
SDGC	0.898	**0.905**	0.861	0.898	0.140	0.897	N/A	0.884	0.041	[[156, 6], [27, 134]]
Perceptron	0.876	0.876	0.876	0.876	0.124	0.876	N/A	0.854	0.069	[[140, 22], [18, 143]]
BNB	0.799	0.800	0.799	0.799	0.201	0.799	0.889	0.814	0.058	([135, 27], [38, 123]]
**Index-TF8M**	**0.989**	**0.989**	**0.989**	**0.989**	**0.011**	**0.989**	N/A	N/A	N/A	**[[160,2],[2,160]]**

In the second step of the pipeline, each model was trained using the same dataset of 537 TRUE phytocystatins but classification was performed among the four subtypes as I1, I2, IwI and II. As observed in the previous step, when individual classification from eight different models were grouped, an improved result was obtained.

Now a new parameter, obtained by pulling results from the 12 best machine learning models (Table 2), was called Phytocystatin Identification Index (PhyCysID 12M), and offers a comprehensive score to support the identification of potential phytocystatin candidates. The improvement is shown in Table 2, which displays a comparison between the individual metrics of each model and the metrics for PhyCysID 12M. It can be observed that all metrics for model performance were higher when PhyCysID 12 M was used, with values ranging between 95% and 99% for accuracy, precision, and specificity.

**Table 2:** Model Performance Evaluation Matrices for Phytocystatin Subtype Prediction

Model	Accuracy	Precision	Specificity	Recall	FPR	F1-Score	AUC-ROC	CV Accuracy	CV Error	Confusion Matrix [l1,fp,fp,fp],[fp,l2,fp,fp],[fp,fp,||,fp],[fp,fp,fp,lwl]
**AdaBoost**	**0.913**	**0.916**	0.964	0.913	0.036	**0.911**	0.954	0.891	0.059	[[16, 3, 1, 0], [3, 29, 1, 0], [0, 0, 86, 0], [1, 0, 5, 16]]
**MLPC**	**0.907**	**0.904**	0.968	0.907	0.032	**0.905**	0.966	0.875	0.058	[[15, 4, 0, 1], [3, 28, 0, 2], [0, 0, 86, 0], [1, 1, 3, 17]]
**RF**	**0.901**	**0.901**	0.963	0.901	0.037	**0.898**	0.966	0.878	0.067	[[I6, 4, 0, 0], [3, 28, 1, 1], [0, 0, 86, 0], [1, 2, 4, 15)]
**LDA**	**0.894**	**0.892**	0.958	0.894	0.042	**0.891**	0.970	0.872	0.056	([14, 4, 1, 1], (2, 28, 1, 2), [0, 0, 86, 0], (0, 1, 5, 16)]
**RC**	**0.894**	**0.897**	0.959	0.894	0.041	**0.891**	N/A	0.870	0.054	([14, 5, 1, 0], [2, 29, 1, 1), [0, 0, 86, 0], (0, 3, 4, 15)]
**LR**	**0.888**	**0.886**	0.957	0.888	0.043	**0.885**	0.960	0.848	0.059	[[15, 4, 1, 0], [2, 28, 1, 2), [0, 0, 85, 1], [0, 3, 4, 15)]
**Bagging**	**0.876**	**0.881**	0.952	0.876	0.048	**0.871**	0.968	0.880	0.057	[[13, 6, 1, 0], [4, 28, 1, 0), [0, 0, 86, 0], [0, 3, 5, 14)]
**GB**	**0.870**	**0.871**	0.951	0.870	0.049	**0.866**	0.954	0.878	0.050	([15, 2, 1, 2], [4, 23, 1, 5), [0, 0, 86, 0], (1, 0, 5, 16)]
**Perceptron**	**0.870**	**0.881**	0.956	0.870	0.044	**0.864**	N/A	0.840	0.056	([20, 0, 0, 0], [8, 21, 1, 3), [0, 0, 86, 0], [3, 3, 3, 13)]
**SGDC**	**0.870**	**0.872**	0.952	0.870	0.048	**0.865**	N/A	0.853	0.047	[[11, 6, 1, 2], [1, 27, 1, 4), [0, 0, 85, 1], [0, 1, 4, 17]]
**GNB**	**0.863**	**0.875**	0.958	0.863	0.042	**0.867**	0.968	0.829	0.072	[[14,6, 0, 0], [3, 28, 0, 2), [0, 0, 79, 7], (1, 2, 1, 18)]
**K-NN**	**0.857**	**0.852**	0.941	0.857	0.059	**0.850**	0.934	0.845	0.064	[[15, I, 2, 2], [4, 25, 2, 2), [0, 0, 86, 0], [1, 2, 7, 12)]
Decision T	0.839	0.835	0.946	0.839	0.054	0.835	0.843	0.827	0.077	[[I4, 2, 1, 3], [9, 22, 0, 2), [0, 0, 86, 0], [0, 6, 3, 13)]
BNB	0.832	0.844	0.945	0.832	0.055	0.836	0.958	0.761	0.082	([17, 3, 0, 0], [3, 26, 1, 3), [1, 6, 76, 3], [2, 3, 2, 15]]
QDA	0.826	0.824	0.943	0.826	0.057	0.822	0.920	0.853	0.074	([13, 6, 1, 0], (5, 24, 0, 4], [0, 0, 85, 1], (1, 8, 2, 11)]
PhyCysID 12 M (training)	0.957	1	0.993	0.965	0.007	0.961	N/A	N/A	N/A	**[[19,0,0,1],[0,31,0,1],[0,1,85,0],[1,0,0,20]]**
PhyCys-ID 12 M (all)	1	0.950	0.991	0.961	0.009	0.955	N/A	N/A		**[[62,1,0,3],[0,104,0,2],[1,4,282,1],[3,1,0,66]]**

### 3.1 Study-Case 1: Testing the Ability to Identify Non Phytocystatin Sequences

Initially, to assess the specificity of our classification pipeline, we tested the sequence of monellin, a small sweet-tasting protein that shares structural similarities with phytocystatins. When monellin peptide sequence was analyzed using PhyCysID, it did not progress beyond the initial stage, being classified as FALSE by five of the eight models in the Index-TF8M, correctly distinguishing it from true members of the family. This result highlights PhyCysID ability to discern subtle yet functionally relevant sequence differences. Consequently, the positioning of the monellin sequence as an outgroup in the phylogenetic tree (Figure 2), rather than clustering with phytocystatins sequences of Eudicots species to which it belongs, demonstrates the high degree of sequence divergence may not represent a functional cysteine proteinase inhibitor, despite its convergent structural features.

### 3.2 Study-Case 2: Testing the Ability to Correctly Classify among Phytocystatin Classes

In this assessment, the sequences of seven phytocystatins from *Arabidopsis* were used to evaluate the performance of each of the 12 machine learning models implemented in PhyCysID. All 12 models were unequivocal in identifying the two type-II cystatin sequences and the intronless subtype I1 cystatin. In contrast, the two type-I cystatin sequences containing introns were identified by 10 and 11 out of the 12 models, respectively. Meanwhile, the two intronless subtype I2 sequences were identified by 11 and 7 models, respectively. Misidentifications made by the models, when they occurred for any of the seven sequences, were highlighted in gray and italicized (Table 3). These findings underscore the relevance of employing multiple machine learning models in the classification of phytocystatin subtypes. Here again, 12 machine learning models are integrated using a majority voting approach. This approach is especially crucial when dealing with closely related subtypes, such as the intronless I1 and I2, which may present overlapping features that challenge single-model classification accuracy and precision.

**Table 3:** Individual Performance of the 12 Models on PhyCysID Classification of *Arabidopsis Thaliana* Phytocystatins

ID	MLPC	LDA	Bagging	RF	AdaBoost	LR	Perceptron	GNB	GB	SGDC	K-NN	RC	Frequency	Final class
**l1_Atha_AT2G31980_1**	l1	l1	l1	l1	l1	l1	l1	l1	l1	l1	l1	l1	12	**l1**
**l2_Atha_AT4G16500_1**	l2	*l1*	l2	l2	l2	l2	*l1*	*l1*	l2	l2	*l1*	*l1*	7	**l2**
**l2_Atha_AT5G47550_1**	l2	l2	l2	l2	l2	l2	*l1*	l2	l2	l2	l2	l2	11	**l2**
**Lwl_Atha_AT2G40880_1**	lwl	lwl	lwl	lwl	lwl	lwl	lwl	lwl	lwl	lwl	*l2*	lwl	11	**lwl**
**lwl_Atha_AT5G12140_1**	lwl	lwl	lwl	lwl	lwl	//	lwl	lwl	lwl	lwl	*II*	lwl	10	**lwl**
**II_Atha_AT3G12490_2**	II	II	II	II	II	II	II	II	II	II	II	II	12	**II**
**II _Atha_AT5G05110_1**	II	II	II	II	II	II	II	II	II	II	II	II	12	**II**

### 3.3 Study-Case 3: Testing PhyCysID on UniProt Protein Dataset

To evaluate the ability of PhyCysID to identify the four different classes of phytocystatins, a test set consisting of 2,314 proteins annotated as phytocystatins in the UniProt database was used. These sequences were retrieved from UniProt by performing a keyword-based search using the terms “cysteine proteinase inhibitor” and “cystatin”, with the results restricted to the taxonomic groups of Monocots and Eudicots.

The retrieved sequences were processed using the pipeline described in Figure 4. As part of the PhyCysID processing, the N-terminal region of each peptide sequence in the FASTA file was trimmed up to the first pair of glycines or the first glycine within the first 50 amino acid residues. Subsequently, a feature table comprising 21 descriptors was generated and used to classify the sequences as true or false phytocystatins using the Index-TF8M. A new filtered feature table was then created, containing only the sequences with an Index-TF8M score equal to or greater than 4. In the next step, the 12 machine learning models from the second stage were applied, enabling PhyCysID to determine the specific phytocystatin class corresponding to each sequence (Table 4).

**Table 4:** Identification of PhyCys Classes in Sequences from UniProt Database

	Number of Models used for Classification									
PhyCys subtype	12	11	10	9	8	7	**6**	5	4	Total
l1	97 (42%)	35(58%)	14(64%)	14(70%)	21(79%)	18(86%)	19(95%)	9(99.1%)	2(100%)	229
l2	140 (29%)	92(48%)	47(57%)	36 (64%)	63 (77%)	40 (86%)	53 (96%)	17 (99.8%)	1 (100%)	489
Lwl	120 (17%)	155(38%)	136 (57%)	61 (65%)	67 (74%)	110 (89%)	60 (98%)	15 (99.7)	2 (100%)	726
II	676 (78%)	63 (85%)	29 (88%)	22 (91%)	28 (94%)	29 (97%)	20 (99.7%)	2 (99.9%)	1 (100%)	870
Total	1035	351	226	133	179	197	152	43	5	2314

The classification of phytocystatin sequences present in the UniProt database shows the distribution of sequence classifications across the four phytocystatin classes, detailing the number of machine learning models that supported the classification of sequences in each class. It is possible to verify that the percentage of sequences identified in each class as the stringency threshold—defined by the minimum number of supporting models—was gradually lowered. For class II phytocystatins, 95% of the sequences were identified by 10 to 12 models, indicating high sensitivity for this class. In the remaining classes, 95% to 99% of the sequences were identified by at least 6 out of the 12 models, also reflecting robust classification performance. It is also observed that among the sequences retrieved from UniProt, there is a higher frequency of cystatins derived from intron-containing genes, namely those belonging to subtypes IwI and II.

## 4 Conclusion

The development of PhyCysID represents a significant advancement in the application of machine learning approaches for the identification and classification of cystatins in plants. By leveraging 21 features derived from amino acid composition and structural properties, and integrating a robust ensemble of 12 distinct machine learning models, PhyCysID effectively distinguishes true phytocystatins from unrelated sequences. This two-tiered strategy, initially filtering out false positives and subsequently classifying valid sequences into four predefined phytocystatin classes, ensures high accuracy and reliability across diverse datasets.

After testing the ability of PhyCysID to identify true phytocystatins and correctly classify them using two controlled scenarios (tailored datasets), the performance of PhyCysID was rigorously evaluated using a curated large dataset comprising 2,314 phytocystatin-like sequences retrieved from UniProt, encompassing both Monocot and Eudicot species. The pipeline demonstrated high sensitivity and specificity, particularly for class II phytocystatins, which were consistently identified by the majority of models. Similarly, classes I1, I2, and IwI were classified with strong consensus across multiple classifiers, with more than 96% of the sequences in each group supported by at least six out of 12 models. These results reflect the robustness and generalizability of PhyCysID across phylogenetic lineages.

In addition to its high predictive performance, PhyCysID offers practical advantages in usability and computational speed. The web server implementation enables both single-sequence and batch submissions, delivering classification results in under 15 s. Moreover, the entire pipeline, from sequence preprocessing and feature extraction to model prediction and result generation, was designed with reproducibility and scalability in mind. Outputs include comprehensive reports on feature importance, model performance, and hyperparameter settings, ensuring transparency and facilitating downstream analyses.

PhyCysID provides an accessible tool for plant molecular biologists, bioinformaticians, and geneticists interested in the study of cysteine protease inhibitors. It supports accurate annotation of phytocystatin genes in newly sequenced genomes and transcriptomes. It can be readily adapted to accommodate future discoveries and expanded training datasets. By combining biological insight with machine learning precision, PhyCysID contributes to a deeper understanding of the structure–function relationships and evolutionary aspects of phytocystatins.

## Supplementary Material









## Data Availability

PhyCysID server is freely available at https://www.ufrgs.br/labec/phycysid.

## Notes

Supplementary File 1—Feature performance by different machine learning models (XLS)
Supplementary File 2—FASTA files containing phytocystatin and unrelated sequence for model training and testing (TXT)
Supplementary File 3—Table from the grid-search to find best hyperparameters for each machine learning model (XLSX)
Supplementary File 4—Compressed file containing the python code used to implement the PhyCysID (ZIP)
